# Molecular Epidemiology of Seal Parvovirus, 1988–2014

**DOI:** 10.1371/journal.pone.0112129

**Published:** 2014-11-12

**Authors:** Rogier Bodewes, Rebriarina Hapsari, Ana Rubio García, Guillermo J. Sánchez Contreras, Marco W. G. van de Bildt, Miranda de Graaf, Thijs Kuiken, Albert D. M. E. Osterhaus

**Affiliations:** 1 Department of Viroscience, Erasmus MC, Rotterdam, the Netherlands; 2 Seal Rehabilitation and Research Centre, Pieterburen, the Netherlands; 3 Viroclinics Biosciences BV, Rotterdam, the Netherlands; 4 Research Center for Emerging Infections and Zoonoses, University of Veterinary Medicine, Hannover, Germany; 5 Artemis One Health, Utrecht, the Netherlands; University of Kansas Medical Center, United States of America

## Abstract

A novel parvovirus was discovered recently in the brain of a harbor seal (*Phoca vitulina*) with chronic meningo-encephalitis. Phylogenetic analysis of this virus indicated that it belongs to the genus *Erythroparvovirus*, to which also human parvovirus B19 belongs. In the present study, the prevalence, genetic diversity and clinical relevance of seal parvovirus (SePV) infections was evaluated in both harbor and grey seals (*Halichoerus grypus*) that lived in Northwestern European coastal waters from 1988 to 2014. To this end, serum and tissue samples collected from seals were tested for the presence of seal parvovirus DNA by real-time PCR and the sequences of the partial NS gene and the complete VP2 gene of positive samples were determined. Seal parvovirus DNA was detected in nine (8%) of the spleen tissues tested and in one (0.5%) of the serum samples tested, including samples collected from seals that died in 1988. Sequence analysis of the partial NS and complete VP2 genes of nine SePV revealed multiple sites with nucleotide substitutions but only one amino acid change in the VP2 gene. Estimated nucleotide substitution rates per year were 2.00×10^−4^ for the partial NS gene and 1.15×10^−4^ for the complete VP2 gene. Most samples containing SePV DNA were co-infected with phocine herpesvirus 1 or PDV, so no conclusions could be drawn about the clinical impact of SePV infection alone. The present study is one of the few in which the mutation rates of parvoviruses were evaluated over a period of more than 20 years, especially in a wildlife population, providing additional insights into the genetic diversity of parvoviruses.

## Introduction

Parvoviruses are small, non-enveloped, viruses with linear, single-stranded DNA genomes of approximately 5 kb. The family *Parvoviridae* comprises two subfamilies: *Parvovirinae* and *Densovirinae*. Members of the *Parvovirinae* infect vertebrates, while members of the *Densovirinae* infect invertebrates [1]. At present, eight genera have been recognized by the International Committee on Taxonomy of Viruses (ICTV) within the subfamily *Parvovirinae*: *Amdoparvovirus*, *Aveparvovirus*, *Bocaparvovirus, Copiparvovirus, Dependoparvovirus*, *Erythroparvovirus, Protoparvovirus* and *Tetraparvovirus* [2], [3].

The genus *Erythroparvovirus* currently consists of six species, *Primate erythroparvoviruses 1–4, Rodent erythroparvovirus 1 and Ungulate erythroparvovirus 1* [2], [3]. Human parvovirus B19 or *Primate erythroparvovirus 1 *is the best studied member of this genus. It is prevalent worldwide; more than 15% of pre-school children, 50% of younger adults and 85% of the elderly have serologic evidence of infection based on data from Australia, Denmark, England, Japan, and the USA [4]. Infection by human parvovirus B19 can be asymptomatic or symptomatic, with clinical manifestations ranging from mild erythema infectiosum (fifth disease) in healthy children to chronic arthropathy in adults, transient aplastic crisis in patients with underlying hematologic disease, and hydrops fetalis [4]–[6]. In addition, involvement of human parvovirus B19 in encephalitis and other neurologic diseases was suggested by serology and viral DNA detection by PCR of the cerebrospinal fluid of patients [7]. Other members of the genus *Erythroparvovirus*; simian parvovirus, rhesus macaque parvovirus, and pig-tailed macaque parvovirus (*Primate erythroparvoviruses 2, 3, 4*), are associated with anemia in the respective non-human primates [8], [9]. Chipmunk parvovirus (*Rodent erythroparvovirus 1*) and bovine parvovirus type 3 (*Ungulate erythroparvovirus 1*) are also members of this genus and were identified in apparently healthy animals [10], [11].

Recently, a novel parvovirus, tentatively called seal parvovirus (SePV), was identified in the brain and lungs tissues of a young harbor seal (*Phoca vitulina*) with severe chronic neurological signs [12]. Phylogenetic analysis suggested that this virus belonged to the genus *Erythroparvovirus*. Histological examination of the brain of the infected seal confirmed the presence of a chronic non-suppurative meningo-encephalitis, while other causes of brain disease, such as neoplasia, physical trauma, and infections with herpesvirus or morbillivirus, were excluded. *In situ* hybridization showed that the novel SePV DNA was present in the Virchow-Robin spaces and in the brain parenchyma adjacent to the meninges of the seal [12]. This discovery showed that parvoviruses indeed can enter the brain parenchyma.

During a previous study using tissue samples collected from necropsies in 2008–2012, SePV DNA was detected in only two out of 94 additional seals [12]. In the present study, this prevalence study was extended using tissues and sera collected from both harbor and grey seals (*Halichoerus grypus*) from Northwestern European coastal waters from 1988 to 2014. In addition, we investigated the genetic diversity, estimated nucleotide substitution rates and evaluated the clinical relevance of SePV infections.

## Materials and Methods

### Ethics statement

Samples of seals used in the present study were provided by the Seal Research and Rehabilitation Centre (SRRC), and the SRRC provided permission to the Department of Viroscience, Erasmus Medical Centre to use the samples for the present study. Admission and rehabilitation of wild seals at the SRRC is permitted by the government of the Netherlands (application number FF/75/2012/015). In the present study, only samples were used that were collected from dead seals or blood samples that were collected for routine diagnostics during rehabilitation. These samples were collected from seals that were either found dead in the wild by the SRRC, died at the SRRC despite intensive care or were euthanized at the SRRC due to the presence of severe clinical signs in the absence of any indication of future recovery. In case of euthanasia, this was performed with T-61 (0.3 ml/kg) after sedation as described previously [13]. Tissue samples that were used in the present study have been described previously [12], [14]–[17].

### Sample collection

Blood samples were collected from harbor (n = 131) and grey seals (n = 69) of different age groups rehabilitated from 2002–2014 at the SRRC, Pieterburen, the Netherlands and after clotting, samples were centrifuged briefly and serum was stored at −20°C until further processing. Samples of spleen tissue (n = 110) were collected from both harbor seals (n = 99) and grey seals (n = 11) that died during outbreaks of phocine distemper virus (PDV) among seals of Northwestern Europe in 1988 and 2002 [16], [18] and in-between years (1988–2002) and stored at −80°C until further processing. An overview of samples used in the present study is listed in **Table S1**. Seals were defined into three different age categories; pups (estimated to be less than two months of age), juveniles (estimated to be between two and twelve months of age) and (sub)adults (estimated to be older than one year of age).

### SePV DNA detection

In previous studies of humans infected with parvovirus B19, viral DNA could not be detected in blood samples, but was detected in tissues such as bone marrow, tonsils, synovium, and lymphoid tissues [19], . Since replication of SePV was observed *in vitro* in seal bone marrow cells, this might be also applicable for SePV [12]. However, since bone marrow samples were not available, spleen tissue samples were selected. These samples were homogenized in 1 ml Hank's minimal essential medium (HMEM) containing 0.5% lactalbumin, 10% glycerol, 200 U/ml penicillin, 200 µg/ml streptomycin, 100 U/ml polymyxin B sulfate, 250 µg/ml gentamycin, and 50 U/ml nystatin (ICN Pharmaceuticals) (transport medium) using a Fastprep-24 Tissue Homogenizer (MP Biomedicals) and centrifuged briefly. Total nucleic acids were extracted from serum (50 µl) and homogenized spleen tissue (200 µl) using the High Pure Viral Nucleic Acid kit (Roche) according to the instructions of the manufacturer. Samples were screened for SePV DNA using a real-time PCR targeting the VP1 gene as described previously [12]. Each sample was tested at least in two independent experiments and to avoid false positive samples due to cross-contamination during mass necropsies, only samples that yielded a cycle threshold (Ct) value below 35 were considered positive.

### Sequencing of the partial genome of SePV variants

On samples that tested positive by real-time PCR, additional specific PCRs were performed to amplify the partial NS (924 bp) and the complete VP2 gene. The partial NS gene was amplified with forward primer TAGAATGGCTTGTGCGGTGT and reverse primer GTGGGTTTCAATGGCCTACT. The complete VP2 gene was amplified using two partial overlapping primer sets, with forward primer TTGCCGGCCATCTCGTCGTA and reverse primer AGCCTGTCCTTCATCTGACC targeting the 5′end of the gene and forward primer TCTCAGGCTCAATGGCGTAG and reverse primer GTGAAGCTTTATTTTTGGGCAC targeting the 3′ end of the gene. PCR products were separated by gel electrophoresis, bands of the appropriate size were extracted using the MinElute Gel Extraction Kit (Qiagen) and cloned using the TOPO TA Cloning Kit for Sequencing (Invitrogen). Multiple clones were sequenced with an ABI Prism 3130xl genetic analyzer (Applied Biosystems). For sequence analysis, a consensus sequence was deduced from at least three colonies for each sample.

### Phylogenetic analysis, estimation of substitution rates and selection pressure

Obtained nucleotide sequences of the complete VP2 and partial NS genes of SePV variants were aligned with various other viruses belonging to the genus *Erythroparvovirus* using MAFFT7 [21]. Subsequently, a maximum likelihood phylogenetic tree was built in MEGA6 [22] with 500 bootstrap values using the Jukes Cantor model, which was selected by analysis with jModeltest 2.1.3 [23].

The number of nucleotide substitutions per site per year was estimated with a Bayesian Markov Chain Monte Carlo (MCMC) method implemented in the BEAST 1.7 package [24]. Dates of sequences were used in combination with the HKY substitution model and a log-normal relaxed clock. The analysis was conducted using a time-aware linear Bayesian skyride coalescent tree prior [25]. Three independent MCMC analyses were performed for 10 million states. These analyses were combined and analyzed with Tracer, version 1.5. Uncertainty in parameter estimates was reported as the 95% highest posterior density (HPD). The selective pressures on the NS and VP2 genes were assessed by calculating the ratio between non-synonymous substitutions (dN) and synonymous substitutions (dS) per site using the single likelihood ancestor counting (SLAC) method implemented in HyPhy platform accessed via the DataMonkey web-server (www.datamonkey.org) [26].

### Analysis of the clinical relevance of SePV infection

The clinical or pathological reports of seals that tested positive for SePV DNA were retrospectively analyzed. Available information included age group (pups <2 months of age, juveniles >2 months and <1 year of age, (sub)adults >1 year of age), sex, gross pathology results and possible cause of death (only for tissue samples), and hematology results (only for the serum samples). Since in a previous study co-infection with herpesvirus was detected in most cases, samples positive for SePV DNA were tested for the presence of co-infection with herpesvirus using a degenerate nested pan-herpesvirus PCR targeting the conserved DNA polymerase gene, as described previously [27]. PCR products with the appropriate band size were sequenced and obtained sequences were analyzed by BLAST search to determine which herpesvirus was detected.

## Results

### Prevalence of SePV infection

Out of 200 seal serum samples from 2002–2014 analyzed, only one sample had a Ct-value <35 (0.5%), which was the serum of a grey seal from 2006 (rehabilitation number: HG 06-130). In addition, in 9 out of 110 spleen tissue samples, the Ct-value was lower than 35 (8%). Of those nine samples, two were collected from harbor seals that had died during the 1988 and seven during the 2002 PDV outbreaks (six harbor seals and one grey seal). Combination of the data obtained in the present study with data from a previous study [12], resulted in a SePV prevalence in spleen samples of 6.9% for harbor seals (based on in total 174 animals) and 9.1% for grey seals (n = 11) (**Figure S1,** **Table 1**).

**Table 1:** Overview of characteristics of seals with SePV.

Sample ID	Sex	Age	Sample	Location of stranding^1^	Year	Ct-value SePV	SePV PCR confirmation	Co-infection with PDV^2^	Co-infection with PhHV-1	Pathological diagnoses and/or major clinical signs
PV880823.6	NI^3^	NI	spleen	UK	1988	27.8	+	NI	-	NI
PV880927.20	NI	NI	spleen	NL	1988	15.4	+	NI	-	NI
HG020628.02	F	(sub)adult	spleen	NL	2002	33.7	+	-	-	Acute non-infectious cause of dead (possible drowning)
PV020628.13	M	(sub)adult	spleen	NL	2002	29.4	+	-	+	Intestinal volvulus
PV020718.12	F	juvenile	spleen	NL	2002	19.9	+	+	+	Extensive, acute, severe, interstitial pneumonia
PV020719.08	F	(sub)adult	spleen	NL	2002	34.1	+	+	+	Extensive, acute, severe, interstitial pneumonia
PV020719.09	M	(sub)adult	spleen	NL	2002	24.3	-	+	-	Extensive, acute, severe, interstitial pneumonia
PV020919.03	F	(sub)adult	spleen	NL, Terschelling	2002	25.0	-	+	-	Euthanized due to presence of severe neurological signs, PDV detected in the brain
PV021004.1	M	(sub)adult	spleen	NL, Vlieland	2002	28.0	-	-4	-	Emaciation; diffuse, subacute, marked, purulent bronchopneumonia
HG06-130	F	juvenile	serum	NL, South-Holland	2006	28.6	+	-	-	Several wounds on flippers and skin lesions suggestive of pox virus infection
PV12-410^5^	M	juvenile	serum and spleen	NL, Ameland	2012	29.6	+	-	-	Chronic, mild, non-suppurative meningo-encephalitis
PV121216.01^5^	F	juvenile	spleen	NL, Terschelling	2012	21.1	+	-	+	Acute, moderate necrotizing bronchitis and bronchiolitis and acute interstitial pneumonia

1 :UK: United Kingdom, NL: the Netherlands.

2 : based on results described previously [14].

3 : NI: no information available.

4 : No PDV was detected [14], although observed lesions were highly suggestive of infection with PDV.

5 : samples from a previous study [12].

### Genetic analysis of SePV

**Figure 1:**
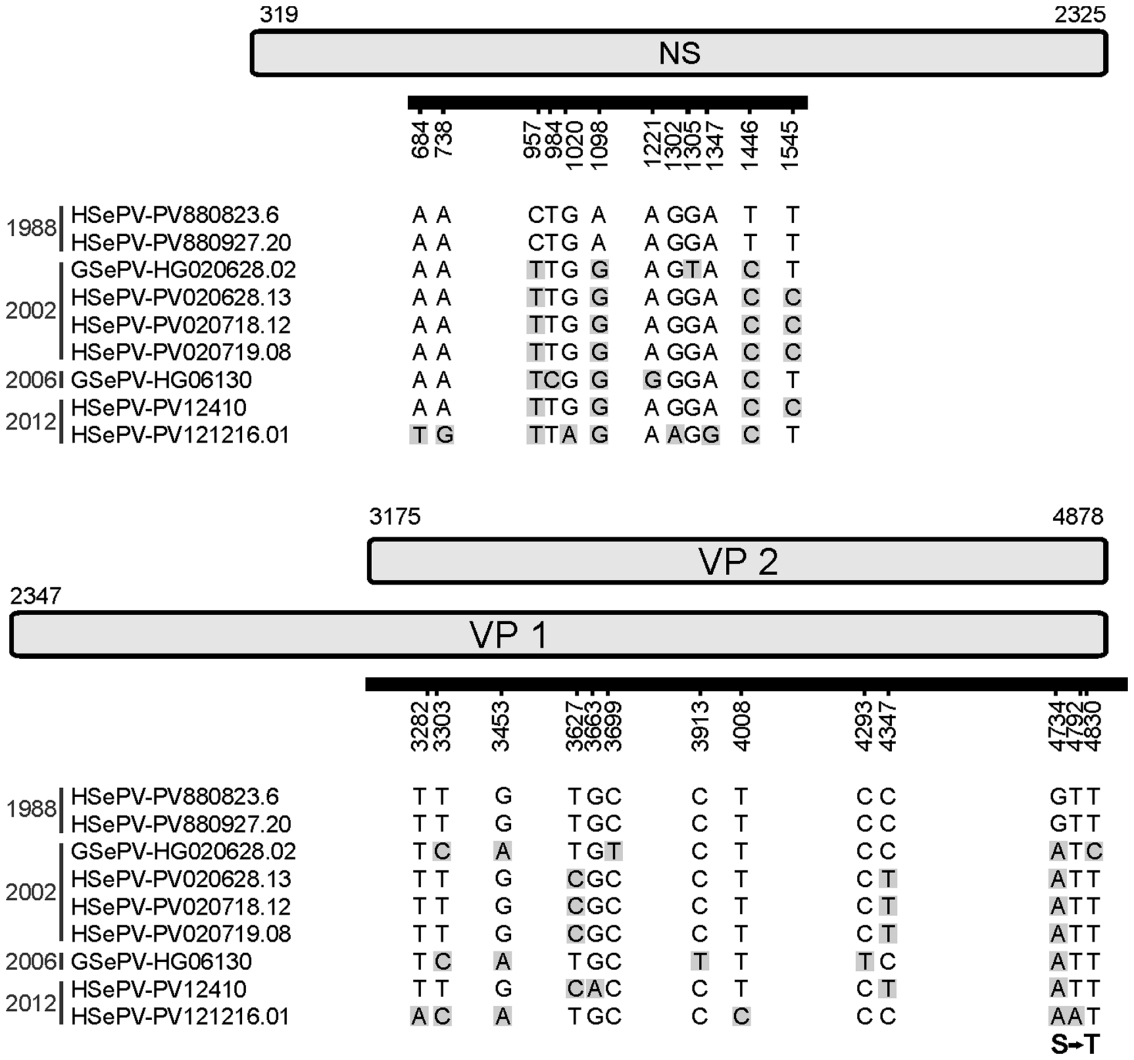
Variation in SePV sequences. Schematic overview of the partial NS gene and the complete VP2 gene that was amplified of 9 SePV variants. The consensus sequence of each virus was compared to the oldest sample available (HSePV-PV880823.6). Locations with nucleotide mutations are indicated in grey. Only in sample HSePV-121216.01, a nucleotide mutation resulted in an amino acid substitution (S540T).

Out of 12 samples in which SePV DNA was detected by real-time PCR (including samples from a previous study [12]), the partial NS (Genbank accession numbers: KM252699-KM252706) and the complete VP2 gene (Genbank accession numbers KM252691-KM252698) could be amplified in only 9 samples, possibly due to fragmentation of the DNA or inhibiting factors present in the other samples. Analysis of the obtained nucleotide sequences showed that SePV from 1988 to 2012 displayed minimal sequence variation. Using the parvovirus detected in the oldest sample (SePV-PV880823.6) as a reference, 12 (1.3%) variant nucleotide positions were present in the partial NS gene (**Figure 1**), of which ten were transitions and two were transversions, and all 12 were synonymous. At three nucleotide positions (positions 957, 1098, and 1446) mutations were fixed in the viruses detected after 1988. All the nucleotide substitutions occurred in the third codon base. Compared to the viruses detected in 1988, the virus with the highest number of nucleotide substitutions was SePV-PV121216.01, which was detected in 2012 (**Figure 1**). Estimated nucleotide substitution rates of the partial NS were 2.00×10^−4^ (HPD, 1.36×10^−5^–4.04×10^−4^) nucleotide substitutions per site per year.

In the complete VP2 gene, 13 (0.76%) variant nucleotide positions were present, including 11 transitions and 2 transversions. Among those, 11 occurred in the third codon base and 2 in the first codon base. In the SePV detected in a harbor seal (identification number PV121216.01), a mutation at nucleotide position 4792 was present, which resulted in an amino acid change from serine to threonine (amino acid residue 540). For the VP gene, we found nucleotide substitution rates of 1.15×10^−4^ (HPD, 1.43×10^−5^–2.22×10^−4^) nucleotide substitutions per site per year with a dN/dS value of 0.33.

**Figure 2:**
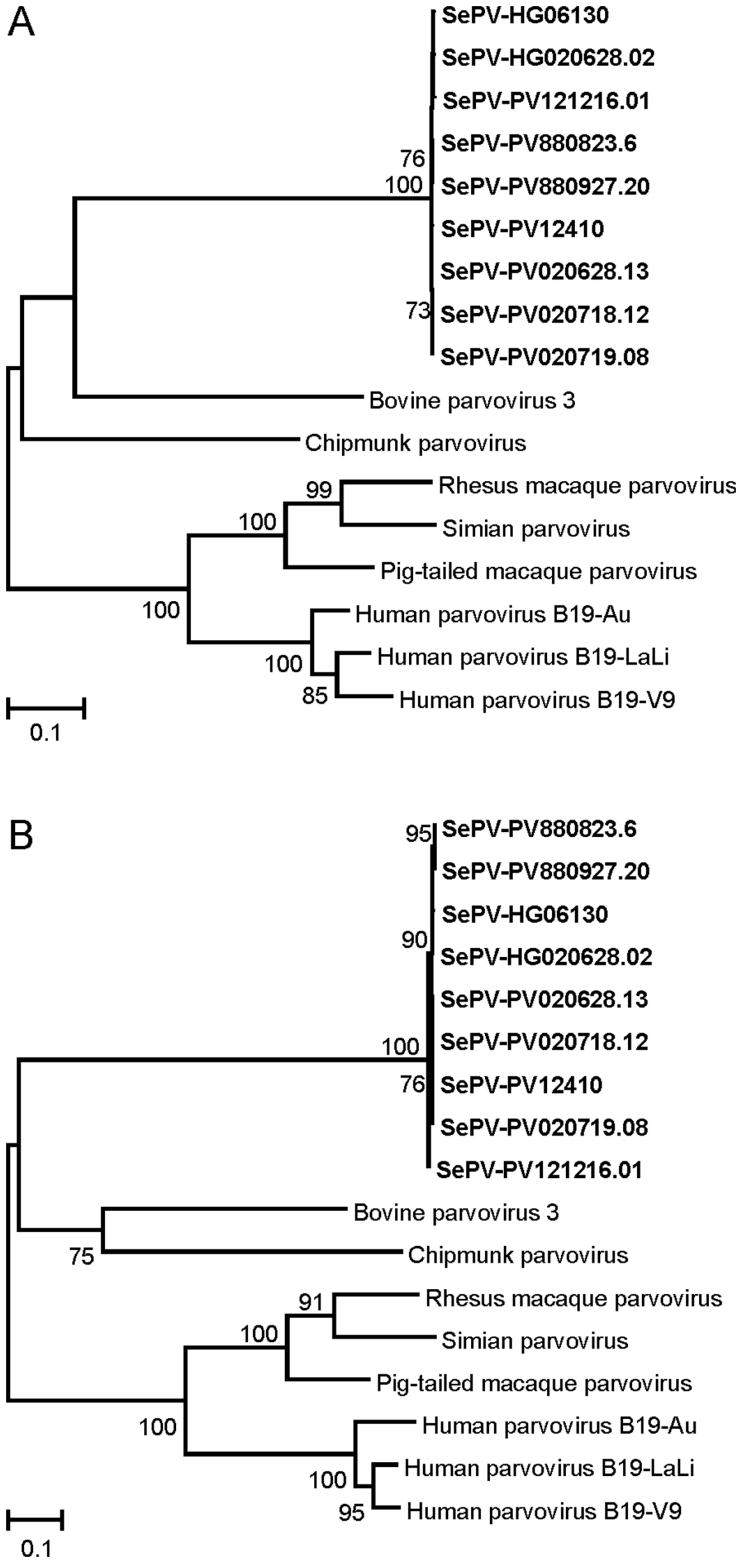
Maximum likelihood tree of the VP2 gene and partial NS1 gene of SePV. Phylogenetic maximum-likelihood tree with 500 bootstrap replicates of the nucleotide sequence of the VP2 genes (**A**) and partial NS genes (**B**) of SePV variants and various viruses of the genus *Erythroparvovirus*. Only bootstrap values >70 are indicated. Genbank accession numbers: SePV-HG06130: KM252691, SePV-HG020628.02: KM252694, SePV-PV121216.01: KM252698, SePV-PV880823.6: KM252692, SePV-PV880927.20: KM252693, SePV12410: KF373759, SePV-PV020628.13: KM252695, SePV-PV020718.12: KM252696, SePV-PV020719.08: KM252697, bovine parvovirus 3: AF406967, chipmunk parvovirus: GQ200736, rhesus macaque parvovirus: AF221122, simian parvovirus: U26342, pig-tailed macaque parvovirus: AF221123, human parvovirus B19-Au: M13178, human parvovirus B19-LaLi: AY044266, human parvovirus B19-V9: AJ249437.

The high similarity of different SePV variants was reflected by phylogenetic and pairwise identity analysis of the NS and VP2 nucleotide sequences. Pairwise identities between SePV NS variants were 99.3% or higher and between SePV VP2 variants 99.5% or higher, while pairwise identities between SePV and other parvoviruses belonging to the genus *Erythroparvovirus* were 51.9% or lower for the NS gene and 50.6% or lower for the VP2 gene (**Figure 2**).

### Evaluation of the clinical relevance of SePV infection

Available clinical and pathology data of the seals from which positive samples were obtained, including two positive seals detected in a previous study [12], showed that SePV DNA was detected in young and (sub)adult seals, but not in pups. Both male and female seals were infected. In both seals in which SePV was detected in the serum, the numbers of red blood cells present in the blood sample was determined. Grey seal HG06-130 had a red blood cell (RBC) count of 3.66×10^12^ cells/liter and harbor seal PV12-410 had a mean RBC value of 3.69×10^12^/liter (ranged from 3.03 to 4.29×10^12^ cells/liter during stay at the SRRC), which were both lower than normal (reference values for a normal RBC count are for both seal species of this age 3.9–5.7×10^12^ cells/liter). Despite a low RBC count, the hemoglobin level of grey seal HG06-130 was 9.2 mmol/liter, which was within the reference values (8.4–14.9 mmol/liter). The mean hemoglobin level of harbor seal 12–410 was somewhat lower than normal (mean value 8.4 mmol/liter, ranged from 6.7 to 9.6 mmol/liter during stay at the SRRC).

Since samples from 1988 and 2002 were collected from seals that had died during PDV outbreaks, the death of at least four seals was related to infection with PDV and the role of SePV could not be evaluated. In one grey seal that died in 2002 (HG020628.02) and in which SePV DNA was detected, the cause of death was non-infectious and no other significant abnormalities were observed upon necropsy of this animal (**Table 1**). One harbor seal that died in 2002 (PV020628.13) had an intestinal volvulus and no significant abnormalities were reported that could be related to a viral infection, although it was positive for phocine herpesvirus-1 (PhHV-1). Besides this seal, PhHV-1 was detected in three additional spleen samples (**Table 1**).

## Discussion

SePV was discovered in 2012 in a young harbor seal with severe chronic meningo-encephalitis [12]. In the present study, we demonstrated that SePV was already circulating among both harbor and grey seals of Northwestern Europe since 1988, although SePV was detected only in a relatively small number of samples.

The partial NS and the complete VP2 gene analyzed in the present study corresponded with highly variable regions of the parvovirus B19 genome [28]. However, sequences of SePV variants analyzed in the present study showed only one amino acid substitution, which was detected in the most recent variant in 2012. This amino acid substitution was present in the VP2 protein (S540T), but since both serine and threonine are polar amino acids it is unlikely that this would have led to substantial conformational change of the VP2 protein. Of interest, there was also only minimal sequence variation between SePV detected in grey seals and harbor seals, similar to what was reported for parapox viruses in two species of pinnipeds and herpesviruses among phocids [29], [30]. These findings suggest that the metapopulation of harbor and grey seals act as a single reservoir for these viruses.

Estimation of nucleotide substitutions rates of parvoviruses has been the focus of multiple studies [31]–[34]. However, the number of studies in which evolutionary dynamics of parvoviruses were evaluated over a period of more than 20 years is limited, especially in a wildlife population [32], [35], [36]. In the present study, we evaluated the annual nucleotide substitutions rates of SePV using samples from 1988 to 2012, which is to our knowledge only the second study performed for viruses of the genus *Erythroparvovirus* over such a long period [35]. However, only a limited number of samples was included in the present study and it could not be excluded that the observed genetic variations were due to polymorphisms of the NS and VP2 genes. In addition, only the partial NS gene was sequenced and observed nucleotide substitution rates might not be representative for the complete NS gene.

Of interest, the relatively low prevalence of this virus in combination with the rapid population growth of both harbor and grey seals from 1988 to 2012 [37] allowed to evaluate genetic diversity of a parvovirus in a population without or with only low herd immunity. Furthermore, harbor seals are generally known to have a limited migration range [38], which minimizes the gene flow of seals that might have differences in susceptibility to this virus and limits the chance of recombination with other parvoviruses from seals of other areas [39]. Although only a relatively low number of different virus variants was studied, observed annual nucleotide substitutions rates were similar to those of other parvoviruses [31]–[34]. Despite this relatively high mutation rate, only one amino acid substitution was detected in the VP2 gene and none in the NS gene, suggesting that natural selection suppressed amino acid changes for both genes. Of interest, also no nucleotide changes were detected in the consensus SePV sequence that was detected in two serum samples collected from seal 12–410 with an interval of five weeks.

SePV was first discovered in a seal with meningo-encephalitis [12], while in the present and previous study SePV was also detected in at least two seals without neurological signs. Since in most cases SePV DNA was detected in seals that were also infected with PDV and/or PhHV-1, the spectrum of clinical signs associated with SePV infection remained unclear. Of interest, results of the present study and a previous study [12] suggested a higher prevalence of SePV during the PDV epizootic in 2002. This might be related to immunosuppression in the seal population following infection with PDV, which predisposes for other viral infections [40]. Anemia or associated signs of low red blood cell counts is one of the manifestations associated with *Erythroparvovirus* infection in humans and non-human primates [6], [8], [9]. In the present and previous study [12], in total two positive samples were identified for which hematology parameters were available. Both seals had relatively low red blood cells counts. However, about 6% of the seals admitted to SRRC have low red blood cells counts upon admission and there are various causes of anemia or low red blood cell counts in seals, such as malnutrition, hemorrhage, chronic disease, and intoxication [41]. Additional studies need to be performed to elucidate the exact impact of SePV infection in both harbor and grey seals.

In summary, the present study showed that SePV has been circulating among both harbor and grey seals from Dutch coastal waters at least from 1988 onwards. Analysis of sequence data suggested that SePV circulating in the Dutch coastal waters showed mutation rates similar to other parvoviruses while this resulted in only one amino acid change. Although the number of virus variants that was analyzed was limited, these results indicate that SePV circulating among seals of the Dutch coastal waters undergoes gradual alteration similar to human parvovirus B19 [35] but with strong negative selection for amino acid changes. These results provide additional insights into the genetic diversity of parvoviruses.

## Supporting Information

Figure S1 **Prevalence of SePV.** Spleen tissues of harbor and grey seals were tested for the presence of SePV DNA. Indicated is the percentage of samples of spleens of seals in which SePV DNA was detected by real-time PCR for all samples of both harbor and grey seals (A), or for specific years for which samples were available from grey seals (B) or harbor seals (C). Numbers above the x-axis represent the number of samples that was tested.
(TIF)

Table S1 **Overview of seal serum and tissue samples used in the present study.**
(DOCX)
