# Supplementary figures and images for: Molecular Epidemiology of Seal Parvovirus, 1988–2014
Source: PLoS One. 2014 Nov 12;9(11):e112129. doi: 10.1371/journal.pone.0112129 (PMC4229121; doi:10.1371/journal.pone.0112129)

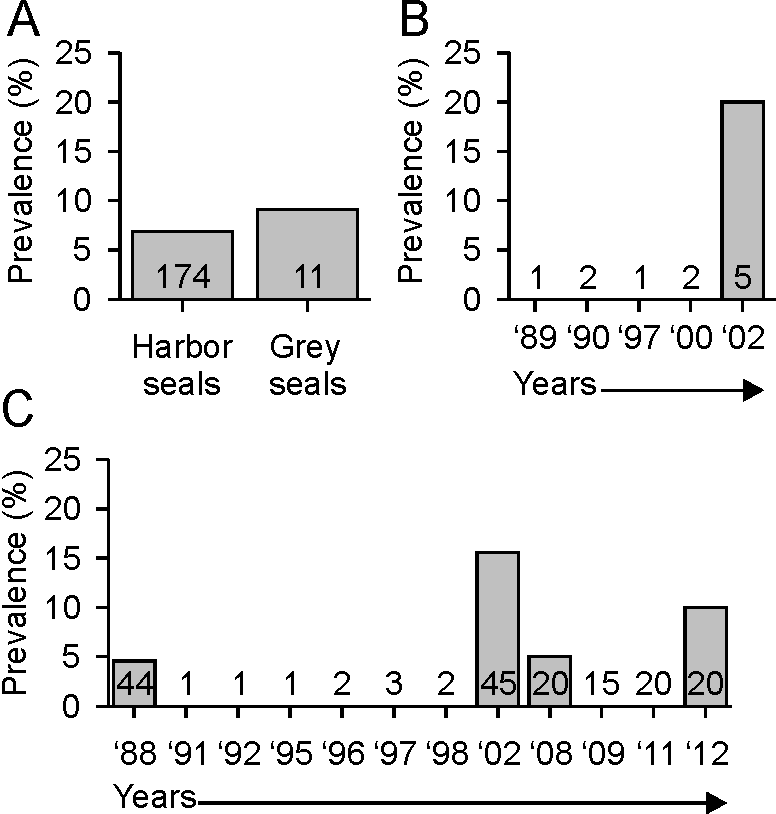

Supplement: Figure S1 — Prevalence of SePV. Spleen tissues of harbor and grey seals were tested for the presence of SePV DNA. Indicated is the percentage of samples of spleens of seals in which SePV DNA was detected by real-time PCR for all samples of both harbor and grey seals (A), or for specific years for which samples were available from grey seals (B) or harbor seals (C). Numbers above the x-axis represent the number of samples that was tested. (TIF) [file pone.0112129.s001.tif]
