# Supplementary material for: Molecular Epidemiology of Seal Parvovirus, 1988–2014
Source: PLoS One. 2014 Nov 12;9(11):e112129. doi: 10.1371/journal.pone.0112129 (PMC4229121; doi:10.1371/journal.pone.0112129)
Supplement: Table S1 — Overview of seal serum and tissue samples used in the present study. (DOCX) [file pone.0112129.s002.docx]

| Year | All seals | Harbor seals | | | Grey seals | | |
| --- | --- | --- | --- | --- | --- | --- | --- |
|  |  | Total | Serum samples | Spleen samples | Total | Serum samples | Spleen samples |
| 1988 | 44 | 44 | 0 | 44 | 0 | 0 | 0 |
| 1989 | 1 | 0 | 0 | 0 | 1 | 0 | 1 |
| 1990 | 2 | 0 | 0 | 0 | 2 | 0 | 2 |
| 1991 | 1 | 1 | 0 | 1 | 0 | 0 | 0 |
| 1992 | 1 | 1 | 0 | 1 | 0 | 0 | 0 |
| 1995 | 1 | 1 | 0 | 1 | 0 | 0 | 0 |
| 1996 | 2 | 2 | 0 | 2 | 0 | 0 | 0 |
| 1997 | 4 | 3 | 0 | 3 | 1 | 0 | 1 |
| 1998 | 2 | 2 | 0 | 2 | 0 | 0 | 0 |
| 2000 | 2 | 0 | 0 | 0 | 2 | 0 | 2 |
| 2002 | 57 | 52 | 7 | 45 | 5 | 0 | 5 |
| 2003 | 9 | 9 | 9 | 0 | 0 | 0 | 0 |
| 2004 | 18 | 14 | 14 | 0 | 4 | 4 | 0 |
| 2005 | 20 | 14 | 14 | 0 | 6 | 6 | 0 |
| 2006 | 15 | 7 | 7 | 0 | 8 | 8 | 0 |
| 2007 | 26 | 10 | 10 | 0 | 16 | 16 | 0 |
| 2008 | 26 | 7 | 7 | 0 | 19 | 19 | 0 |
| 2009 | 11 | 10 | 10 | 0 | 1 | 1 | 0 |
| 2011 | 20 | 18 | 18 | 0 | 2 | 2 | 0 |
| 2012 | 20 | 12 | 12 | 0 | 8 | 8 | 0 |
| 2013 | 17 | 14 | 14 | 0 | 3 | 3 | 0 |
| 2014 | 11 | 9 | 9 | 0 | 2 | 2 | 0 |
| Total | 310 | 230 | 131 | 99 | 80 | 69 | 11 |
